# Functional Outcome of Closed Reduction and Extension Casting in Forearm Fractures in Children

**DOI:** 10.7759/cureus.22389

**Published:** 2022-02-19

**Authors:** Parth Yadav, Mukesh O Phalak, Shivam Patel, Tushar Chaudhari, Abhishek Nair, Sagar Gurnani

**Affiliations:** 1 Orthopedics, Dr. D.Y. Patil Medical College, Hospital, and Research Center, Pune, IND; 2 Orthopedic Surgery, Dr. D.Y. Patil Medical College, Hospital, and Research Center, Pune, IND

**Keywords:** pediatric forearm fractures, conservative management, closed reduction, forearm fractures, extension casting

## Abstract

Introduction

Fractures in children are extremely common scenarios encountered by orthopedicians. Conservative treatment has been the most preferred choice for the management of diaphyseal forearm fractures. Traditionally, pediatric forearm fractures are treated by above elbow plaster cast with the elbow flexed to 90 degrees. The purpose of this study was to evaluate the functional and radiological outcomes of children treated with closed reduction and extension casting for forearm fractures.

Patient and methods

This is a prospective study evaluating the functional and radiological outcomes of 30 children of less than the age of 14 years and without pathological fractures, treated with closed reduction and extension casting for forearm fractures, either both radius and ulna or radius or ulna at middle third level, who reported to the Department of Orthopedics, Dr. D.Y. Patil Medical College, Hospital, and Research Center, Pune, India, between September 2019 and March 2022.

Results

The mean pre-operative angulation in radius (antero-posterior {AP}) was 22.7, radius (lateral {LAT}) was 24.2, ulna (AP) was 31.2, and ulna (LAT) was 29.2. The immediate post-operative angulation of radius (AP) was 0.7, radius (LAT) was 3.2, ulna (AP) was 0.6, and ulna (LAT) was 4.9. Cast status at two weeks, 83.3% had intact cast and 16.7% had loosened casts. Post removal, most patients had a good rotation of motion (ROM) at three and six weeks.

Conclusion

Casting with extended elbow is much better as compared to flexion casting in the hands of a trainee doctor. Furthermore, chances of loss of reduction are negligible in extension casting as compared to flexion casting.

## Introduction

Fractures in children are extremely common scenarios encountered by orthopedic surgeons [1]. In children, forearm fractures account for 25% of all fractures [2]. Some literature evidence suggests that forearm fractures constitute 30-50% of all pediatric trauma [3-6]. Diaphyseal fractures constitute approximately 6% of all other children’s fractures [2]. Children between the age of five to 14 years have the highest fracture rates [7]. Male children less than 16 years of age show 42% more risk of fracture as compared to 27% among girls [2].

Conservative treatment was previously the most preferred choice for the management of diaphyseal forearm fractures; however, now the trend towards surgical management is increasing [8-11]. This trend is attributed to the complications of conservative management such as loss of mobility and re-displacement [12]. However, the management of forearm shaft fractures varies [13]. The conservative treatment method involves the use of cast or closed reduction and cast application. Surgical treatment involves the use of percutaneous intramedullary nails or plate and screws fixation. The most common complication of pediatric forearm fractures is loss of reduction [7]. During follow-up, re-displacement rate ranges from 7% to 27% [14-16]. Identification of potential predictors of re-displacement may improve the effectiveness of cast immobilization and also helps to decide whether surgical intervention is warranted.

Management of both-bone forearm fractures is considered difficult due to inherent instability. Loss of reduction causes a significant psychological, financial, and physiological burden on the patient. Traditionally, pediatric forearm fractures are treated by above elbow plaster cast with the elbow flexed to 90 degrees. Bochang et al. in 2005 suggested that immobilization with the elbow in extension is a viable alternative to traditional flexion casting in forearm fractures, and this extension casting technique has been adopted by various surgeons since the study by Bochang et al. [17].

Closed reduction and casting remain the gold standard treatment for pediatric forearm fractures [15]. Some studies have stated that closed reduction and casting is a viable choice even in cases of 100% displacement of radius and ulna owing to its excellent remodeling capacity in children less than the age of nine years [18]. The exact values of angulation, rotation, and displacement are debatable in the literature [19]. However, it is believed that a fracture closer to the diaphysis has a greater potential for remodeling.

Closed reduction and casting method is adopted worldwide across various institutions but the principle of treatment remains common. The method is carried by sedation/anesthesia, recreation of initial deformity allowing unlocking of fracture, obtaining length, and reduction of the angular/rotational deformity and casting. The said procedure is usually done under sedation in casualty. An appropriate three-point molding of cast is essential for forearm fractures management which includes appropriate padding, sufficient plaster material for molding; it should have less heat generation and should not be too heavy. Cast index is the ratio of the sagittal to coronal width of cast and is considered integral for successful closed management. Kamat et al. suggested significant loss of reduction occurs if a cast index is above 0.7-0.8 [20]. An above-elbow cast for any forearm fractures is recommended for children under four years of age as short arm casts may slip [21,22]. A follow-up during the first three weeks post-reduction is recommended in order to make sure of maintenance of reduction. Holmes et al. stated, in cases of reduction loss, cast may be wedged appropriately, which can be a suitable alternative to restore alignment, but re-reduction or operative intervention may be required [22].

This study aimed to evaluate the functional and radiological outcomes of children treated with closed reduction and extension casting for forearm fractures and to analyze the reduction achieved, angulation at fracture site, and range of motion of elbow joint after removal of cast.

## Materials and methods

A prospective observational study was conducted evaluating the functional and radiological outcomes of children treated with closed reduction and extension casting for forearm fractures who reported to the Department of Orthopedics, Dr. D.Y. Patil Medical College, Hospital, and Research Center, Pune, India, between September 2019 and March 2022. Ethical clearance was obtained from the meeting of the Research and Recognition Committee under the Faculty of Medicine, Dr. D.Y. Patil Medical College, Hospital, and Research Center, Pune (IESC/PGS/2019/98/ 2019). The patient and relatives were explained regarding the condition of the patient and informed consent was taken along with the details of the patient. All investigations and procedures were done when clinically indicated. Pre- and post-operative assessments were performed to assess their functional outcomes.

Inclusion and exclusion criteria

Children with age less than 14 years with either radius or ulna fracture or both radius and ulna fracture at any site were included in the study. Patients with age more than 14 years, children with compound fractures of forearm, and children with pathological fractures were excluded.

Procedure

**Figure 1:**
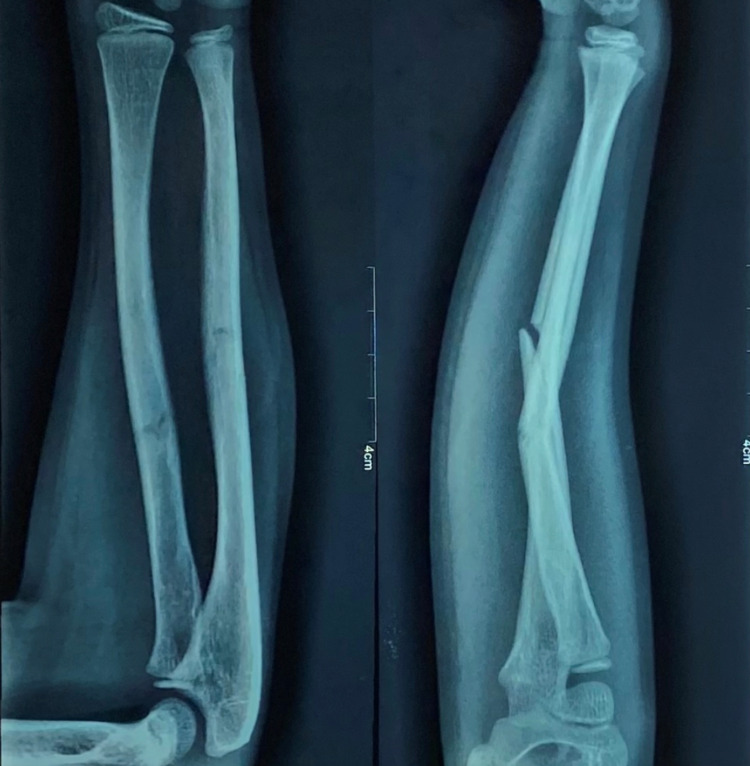
Pre-operative radiograph of a case of radius and ulna shaft fracture

**Figure 2:**
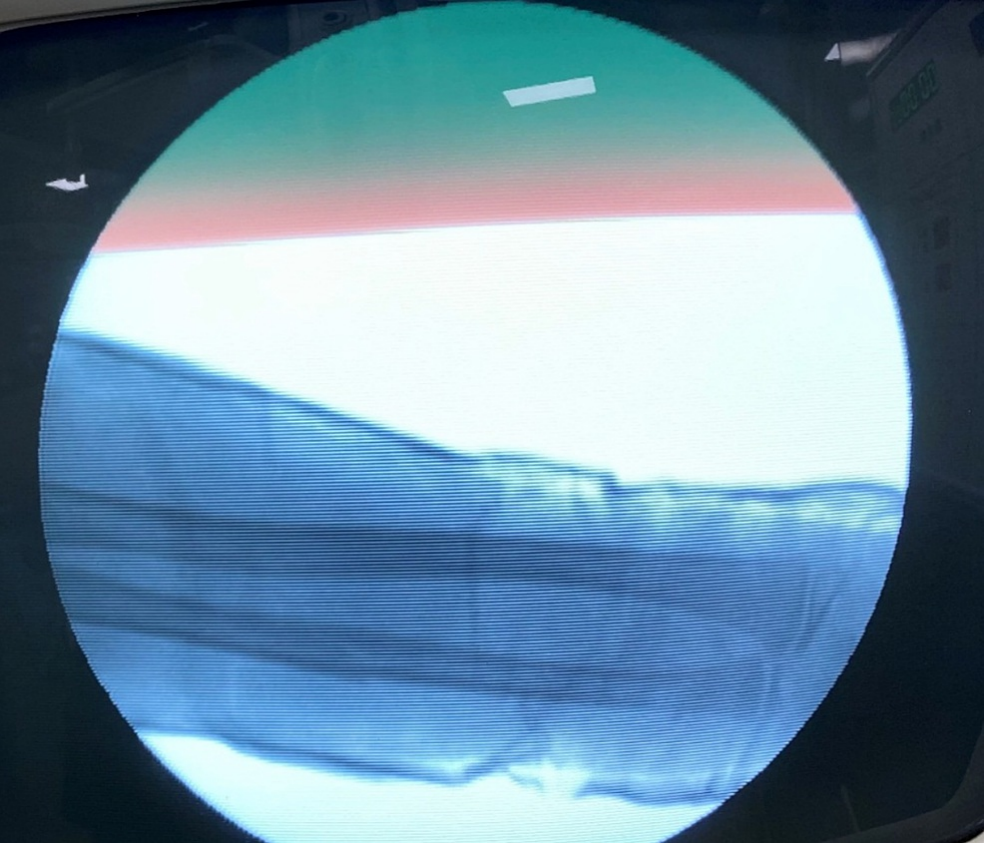
Intra-operative "c-arm" picture antero-posterior view after performing reduction

**Figure 3:**
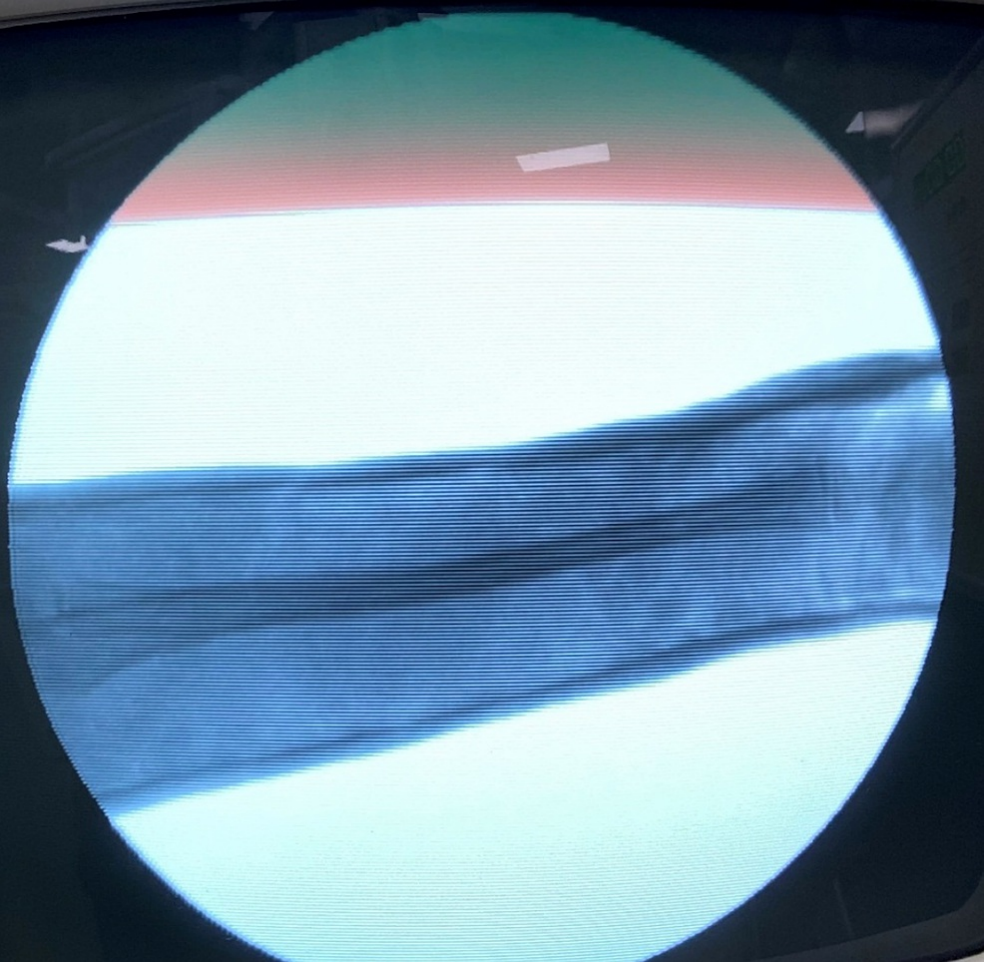
Intra-operative 'c-arm' picture lateral view after performing reduction

Pre-operative antero-posterior (AP) and lateral x-ray were taken (Figure 1). Adequate sedation was given to the patient. Fracture was reduced by the standard technique of axial traction manipulation. Reduction was confirmed radiologically by image intensifier and if found satisfactory, cast application was done (Figures 2, 3).

**Figure 4:**
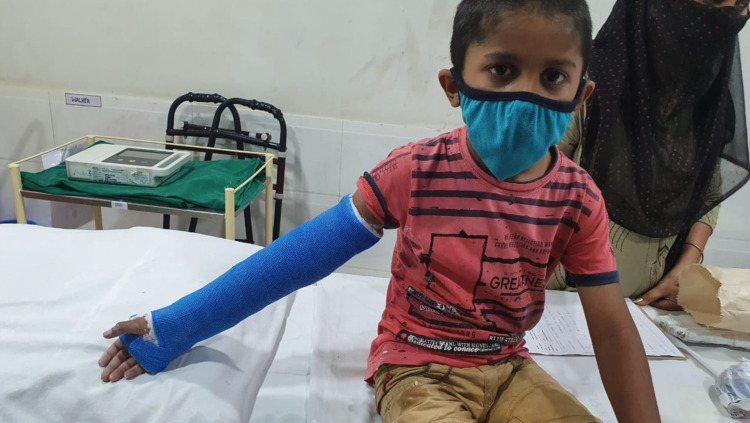
Clinical picture of a child with casting done in extended position

The above-elbow cast was put after proper padding, with elbow joint in extension and forearm in the most stable position (Figure 4). A cast index = 0.7 or less, calculation of which was done by dividing inner sagittal width of cast by inner coronal width of cast at fracture level, decreases fracture re-displacement risk. If reduction was not found to be acceptable, the process was repeated under general anesthesia with the help of a C-arm.

Criteria for analysis

**Figure 5:**
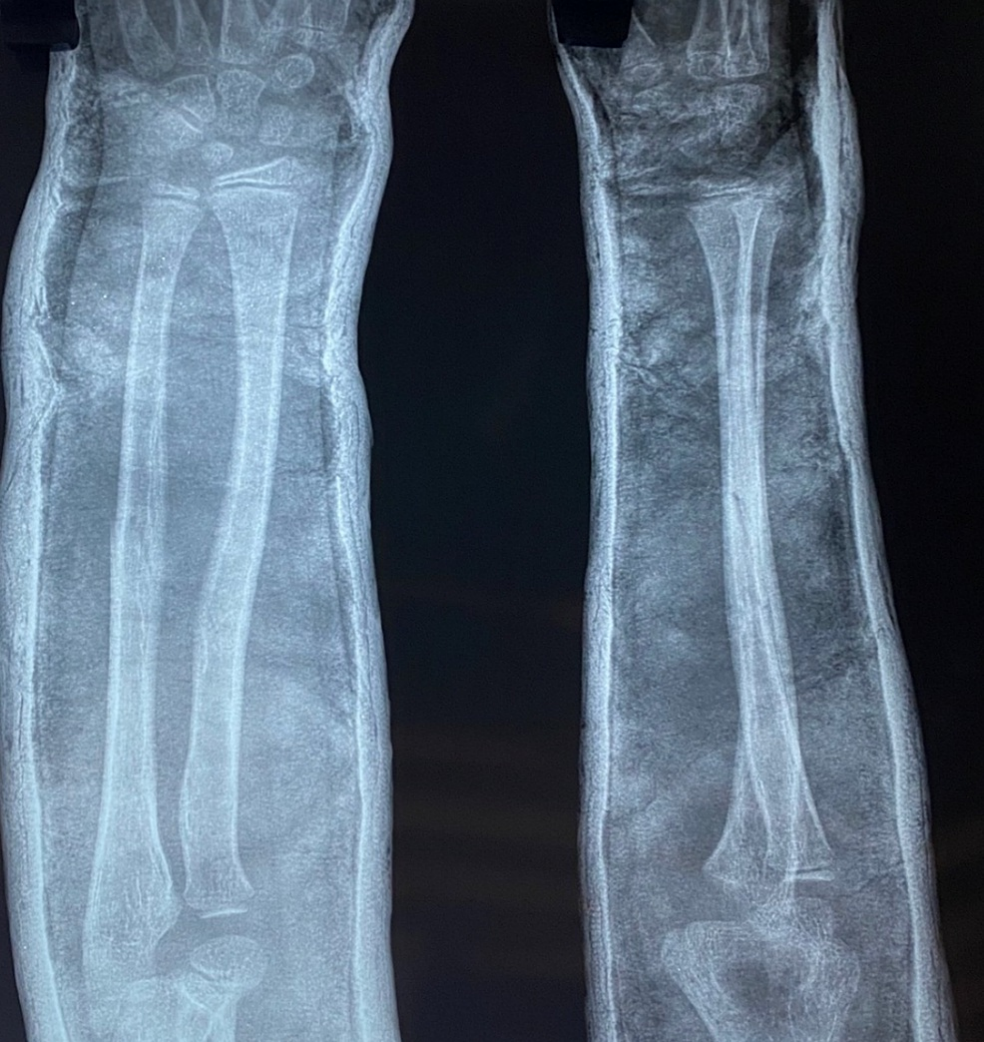
Post-operative radiograph taken two weeks after casting

**Figure 6:**
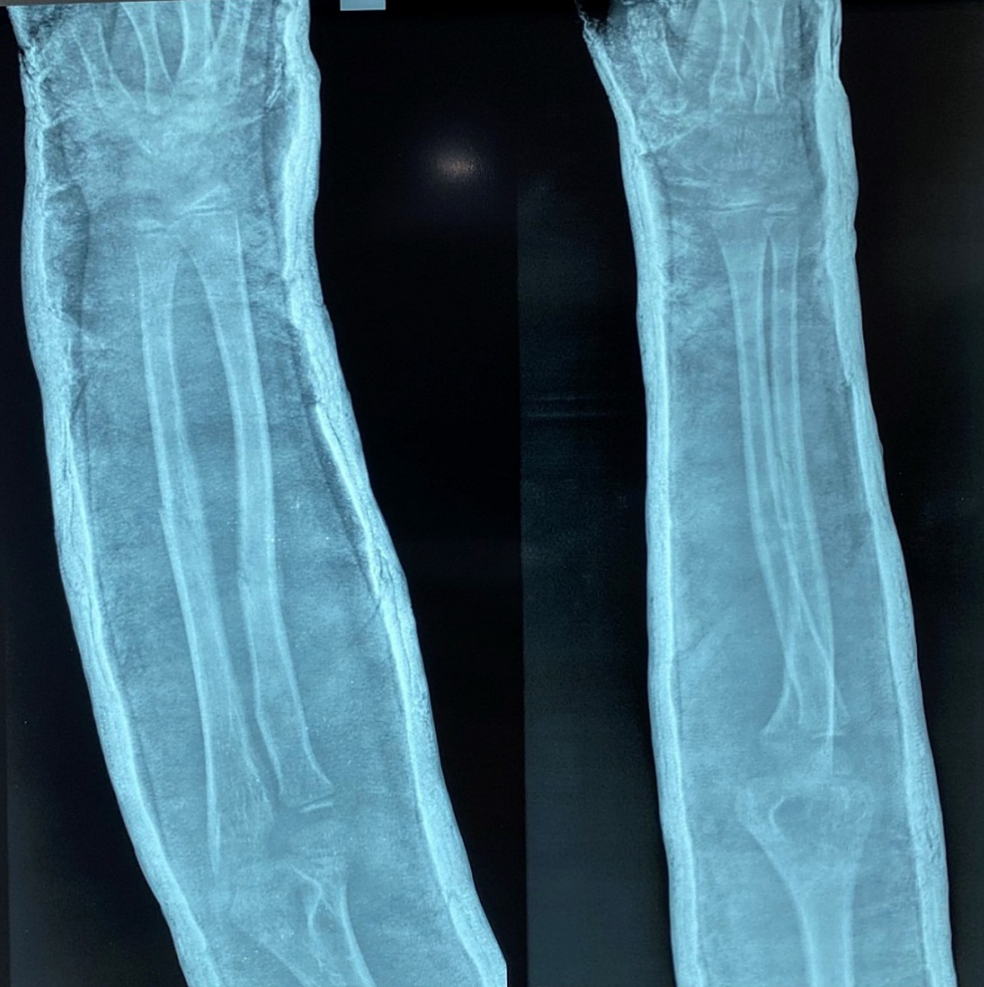
Post-operative radiograph taken four weeks after casting

Antero-posterior (AP) and lateral view x-rays were recorded at two weeks post-reduction (Figure 5) and if no re-displacement was found, the cast was kept for four to six weeks and repeat radiographs were taken at four and six weeks and cast was removed (Figure 6). Radiographs were assessed for any angulation of the union site to assess for malunion. Clinical examination was performed three and six weeks after the removal of cast to assess the movement of the elbow and forearm. Stiffness of the elbow joint was assessed. The results were compared with the results of previously published results of casting of forearm fractures in flexion to determine the efficacy of casting. 

## Results

Pre-operative and immediate post-operative angulation in radius and ulna in AP and lateral views

The mean pre-operative angulation in radius (AP) was 22.7, radius (lateral {LAT}) was 24.2, ulna (AP) was 31.2, and ulna (LAT) Caswas 29.2. The immediate post-operative angulation of radius (AP) was 0.7, radius (LAT) was 3.2, ulna (AP) was 0.6, and ulna (LAT) was 4.9 (Table 1). Out of the 30 patients, cast status at two weeks post-operative was 25 patients (83.3%) had intact cast and five patients (16.7%) had loose (reinforced) cast status (Table 2).

**Table 1:** Pre-operative and immediate post-operative angulation of radius and ulna in AP and lateral views AP: antero-posterior; LAT: lateral

Parameter	Mean (degree)	SD	Minimum (degree)	Maximum (degree)
Pre-operative angulation in radius (AP)	22.7	10.17	2	40
Pre-operative angulation in radius (LAT)	24.2	9.18	8	42
Pre-operative angulation in ulna (AP)	31.2	5.6	20	40
Pre-operative angulation in ulna (LAT)	29.2	6.83	16	42
Immediate post-operative angulation radius (AP)	0.7	1.0	0	2
Immediate post-operative angulation radius (LAT)	3.2	1.9	0	6
Immediate post-operative angulation ulna (AP)	0.6	1.2	0	4
Immediate post-operative angulation ulna (LAT)	4.9	2.9	0	10

**Table 2:** Cast status two weeks post-operative

Status	Number	Percentage
Intact	25	83.3
Loose (reinforced)	5	16.7
Total	30	100.0

Comparison of angulation

The mean pre-operative angulation of radius (AP) was 22.7 and the mean post-operative angulation of radius (AP) was 0.69. This comparison was statistically significant (p<0.001) (Table 3). The mean pre-operative angulation of ulna (AP) was 31.2 and the mean post-operative angulation of ulna (AP) was 0.6. This comparison was statistically significant (p<0.001) (Table 4).

**Table 3:** Comparison of pre- and post-operative AP radius *Paired t-test. **Statically significant. AP: antero-posterior

Parameter	Number	Mean	SD	p-Value
Pre-operative angulation of radius (AP)*	23	22.7	10.17	<0.001**
Post-operative angulation of radius (AP)*	23	0.69	0.20	

**Table 4:** Comparison of pre- and post-operative AP ulna *Paired t-test. **Statically significant. AP: antero-posterior

Parameter	Number	Mean	SD	p-Value
Pre-operative angulation of ulna (AP)*	30	31.2	5.6	<0.001**
Post-operative angulation of ulna (AP)*	30	0.6	0.2	

The mean pre-operative angulation of lateral radius was 24.2 and the mean post-operative angulation of lateral radius was 3.2. This comparison was statistically significant (p<0.001) (Table 5). The mean pre-operative angulation of ulna (lateral) was 29.2 and the mean post-operative angulation of ulna was 4.9. This comparison was statistically significant (p<0.001) (Table 6).

**Table 5:** Comparison of pre- and post-operative lateral radius *Paired t-test. **Statically significant.

Parameter	Number	Mean	SD	p-Value
Pre-operative angulation of radius (lateral)*	23	24.2	9.18	<0.001**
Post-operative angulation of radius (lateral)*	23	3.2	0.4	

**Table 6:** Comparison of pre- and post-operative lateral ulna *Paired t-test. **Statically significant.

Parameter	Number	Mean	SD	p-Value
Pre-operative angulation of ulna (lateral)*	30	29.2	6.83	<0.001**
Post-operative angulation of ulna (lateral)*	30	4.9	0.5	

## Discussion

Forearm fractures account for 30-50% of fractures occurring in the pediatric population. Conventionally, in order to treat these pediatric fractures of the forearm, the technique of closed reduction followed by casting was practiced. Closed reduction and casting remain the gold standard in the management of pediatric forearm fractures owing to their remodeling capabilities in younger patients [15]. Few studies in literature have noted that closed reduction and casting is an excellent treatment option even in 100% displaced radius and ulnar bone in children less than nine years of age. It is assumed that considering the growth and remodeling potential of the bones, even if achieving anatomical reduction is not possible, the residual deformity would get corrected. But, the precise values of displacement, angulation, and rotation, which can be accepted, are controversial in the existing literature.

Bochang et al. have done a study on extended vs flexed elbow casting following closed reduction in children with forearm fractures and have concluded that there was a 17-18% rate of need for re-manipulation when these fractures were managed using a cast in elbow flexion whereas no such need for re-manipulation was seen in elbow extended casts [17]. Walker and Rang stated that in a hospital setting, extension casting proves to be of higher value because of the relative ease of the application of the cast. This is of utmost importance when it is being performed by a single person, often a trainee, thus resulting in reduced chances of reduction loss which reduces the need for re-manipulation as there is no involvement of flexion of the elbow when extension casting was utilized [23]. The study conducted by Babazadeh et al. also showed that extension casting is a better modality of treatment for distal forearm fractures. This is explained by the neutralization of the deforming force of the brachioradialis muscle on the distal part of the radius [24].

With respect to extension casting, the reduced failure would be directly related to the effect of this type of immobilization on the supination forces which act on the proximal fragment of the fracture. Another point that needs to be taken into consideration in cases of flexion casting is that as the swelling of the surrounding soft tissue subsides the fracture fragments tend to become unstable and there is loss of apposition. This is typically seen because of the action of gravity which leads to the production of a fulcrum at the involved site.

Petterwood et al. studied extension casting for both bone forearm fractures for children. They noted that significant residual angulation was present at follow-up in flexion casting patients in comparison to the extended elbow cast patients in both radius and ulna. He concluded that closed reduction and casting with the elbow in extension is a safe and much better technique that maintains reduction of both bone forearm fractures in young patients [25].

Arora et al. studied the role of risk factors and above-elbow cast indices to predict notable re-displacement of fractures of forearm in children managed by closed reduction and casting. They concluded that managing conservatively with the objective of reducing anatomically, mainly in patients showing complete displacement, must be the main approach in closed fractures of forearm in children [26]. Ajmera et al. assessed the rate of re-displacement in pediatric forearm fractures who were treated by cast by calculating the cast index. The authors concluded that there is a sufficient association of cast index in predicting the outcome of pediatric forearm fractures [27]. Holmes et al. reported that, if there is occurrence of re-displacement, cast may be wedged to achieve alignment, but repeat reduction or operative procedure might be needed [22]. Babazadeh et al. stated that flexion casting often does lead to complications such as pressure ulcers typically in the region of the cubital fossa [24].

In our study, out of the 30 patients, the mean age of study participants was 7.3 years with 18 (60%) males and 12 (40%) females. Sports injury was the commonest etiological factor attributing to forearm fractures. Out of the 30 patients, seven had only ulna fracture (23.3%) whereas 23 children had radius and ulna fracture (76.7%) with left side being more involved (53.3%) as compared to the right side (46.7%).

Closed reduction is advised in patients of zero to eight years of age with angulation of fracture more than 100 and malrotation more than 300 [14]. For patients having angulation below 100 and malrotation below 300, applying splint without performing reduction can be accepted. In our study, the mean pre-operative angulation in radius (AP) was 22.70, radius (LAT) was 24.20, ulna (AP) was 31.20, and ulna (LAT) was 29.20 and the immediate post-operative angulation of radius (AP) was 0.70, radius (LAT) was 3.20, ulna (AP) was 0.60, and ulna (LAT) was 4.90. The mean pre-operative angulation of ulna (lateral) was 31.2 and the mean post-operative angulation of ulna (lateral) was 4.9 (p<0.001). The mean pre-operative angulation of lateral radius was 24.2 and mean post-operative angulation of lateral radius was 3.2. (p<0.001).

Literature evidence shows that angulation of 15-200 in the middle one-third fractures of forearm has the propensity to cause significant loss of rotation in the forearm [4]. But, the significance of loss of range of motion (ROM) in clinical outcomes remains debatable. In our study, at three weeks after cast removal, 10 (33.3%) patients showed range of motion of 0-200, 11 (36.7%) patients showed a ROM of 0-300, and nine (30%) patients showed a ROM of 0-450. At six weeks after cast removal, six (20%) patients showed flexion of 0-1250, six (20%) showed flexion 0-1300, six (20%) showed flexion 0-1400,and 12 (40%) showed flexion 0-1450. Six weeks after removal of cast, supination and pronation were also checked. In nine (30%) patients, pronation was 65 degrees whereas in 10 (33.3%) and 11 (36.7%) patients, pronation was 700 and 750, respectively. In eight (26.7%) patients, supination was 700 whereas in 10 (33.3%) and 12 (40%) patients, supination was 750 and 800, respectively.

Cast index is the ratio of sagittal to coronal width of cast and is useful to predict successful management of closed fractures. In our study, out of the 30 patients, cast status at two weeks post-operative was 25 (83.3%) patients had intact cast and five (16.7%) patients had loose (reinforced) cast status [21,27]. In our study, we did not encounter any complications like re-displacement of fracture requiring re-manipulation, stiffness at the elbow joint, pressure ulcers, which can be associated with casting in flexed position.

Limitations of this study must be addressed. We had a limited number of patients in this time frame partly due to the coronavirus disease 2019 (COVID-19) pandemic. A prospective study comparing both flexion and extension casting with a larger sample size needs to be done in order to make either of the methods standardized in clinical practice.

## Conclusions

Closed reduction followed by cast application remains the gold standard in the management of forearm fractures in children as it improves the quality of treatment and reduces the need for re-manipulation. From our study, we conclude that casting with elbow extended can be a better option as compared to flexion casting in the hands of a trainee doctor as compared to the results of flexion casting in the existing literature. More extensive studies with a control group need to be done to establish extension casting as a standard method of treatment.
